# Total Internal Reflection Fluorescence (TIRF) Single-Molecule Assay to Analyze the Motility of Kinesin

**DOI:** 10.21769/BioProtoc.5135

**Published:** 2024-12-20

**Authors:** Tomoki Kita, Shinsuke Niwa

**Affiliations:** 1 Graduate School of Life Sciences, Tohoku University, Miyagi, Japan; 2 Frontier Research Institute for Interdisciplinary Sciences (FRIS), Tohoku University, Miyagi, Japan

**Keywords:** Kinesin, Microtubules, Single-molecule assay, KIF1A, UNC-104

## Abstract

The motile parameters of kinesin superfamily proteins are fundamental to intracellular transport. Single-molecule motility assays using total internal reflection fluorescence (TIRF) microscopy are a gold standard technique for measuring the motile parameters of kinesin motors. With this technique, one can evaluate the velocity, run length, and binding frequency of kinesins on microtubules by directly observing their motility. This protocol provides a comprehensive procedure for single molecule assays of kinesins, including the preparation of labeled microtubules, the measurement of kinesin motility via TIRF microscopy, and the quantification of kinesin motor parameters.

Key features

• Analysis of the motility of kinesin superfamily proteins using TIRF microscopy.

• In vitro reconstitution using purified microtubules and motors.

• Direct measurement of motile parameters of kinesins.

## Background

Intracellular transport is essential for many cellular processes. Kinesin is an ATP-dependent molecular motor that moves along microtubules and facilitates anterograde transport [1]. Organisms have many kinesins, and each kinesin has motile properties specific to its function [2]. Fluorescence microscopy allows us to observe kinesins or their cargo labeled with fluorescent proteins. However, background fluorescence from outside the focal plane can hinder the observation of single fluorophores and the examination of kinesin motility at a single-molecule resolution. In TIRF microscopy, the laser reflects between the coverslip and solution to form an evanescent field that excites the fluorescent protein on the motor [3]. This excitation is restricted to a region typically less than 100 nm in thickness, allowing us to track kinesins with single fluorophores [3].

Using TIRF microscopy, single-molecule parameters of kinesins, such as velocity and run length, have been well studied [4,5]. Disease-associated motile defects of kinesins can be directly measured by this method [6–9]. Additionally, the assay can analyze the extent of kinesin activation [10,11]. Most kinesins are inactivated by autoinhibition to avoid binding to microtubules without cargo [12]. Upon release from autoinhibition, the motor’s landing rate on microtubules increases dramatically, enabling us to observe their activated movements along microtubules [10,11].

In this protocol, we present three methods for single-molecule assays. The first involves modifying microtubules to stabilize them on glass surfaces using biotin-streptavidin interactions and visualize them with fluorescence. The second focuses on observing the motility of individual kinesin molecules, and the third covers their subsequent analysis including velocity, run length, and landing rate. Our comprehensive protocol is well-suited for researchers entering the field of kinesin molecular motors.

## Materials and reagents

**Biological materials**

1. Porcine tubulin; tubulin was purified from porcine brain, as described [13].
2. Kinesin motor of interest; in this protocol, KIF1A(1-393)LZ::mScarletI, UNC-104(1-653)::sfGFP and UNC-104(1-653)(E412K)::sfGFP are used as examples for TIRF assays. Protein was produced as previously described using LOBSTR-BL21(DE3)-RIL *E. coli* (Kerafast, catalog number: EC1002) in the case of KIF1A(1-393)LZ::mScarlet [8] and *Sf9* insect cells in the case of UNC-104(1-653)::sfGFP and UNC-104(1-653)(E412K)::sfGFP [4].

**Reagents**

1. Piperazine-1,4-bis(2-ethanesulfonic acid) (PIPES) (FUJIFILM Wako Pure Chemical Corporation, catalog number: 345-02225)
2. Magnesium chloride (MgCl_2_) (Sigma-Aldrich, catalog number: M2393)
3. Ethylene glycol tetraacetic acid (EGTA) (Nacarai Tesque, catalog number: 15214-92)
4. Potassium hydroxide (KOH) (Nacarai Tesque, catalog number: 28616-45)
5. 2-[4-(2-Hydroxyethyl)-1-piperazinyl]ethanesulfonic acid (HEPES) (Nacarai Tesque, catalog number: 17514-15)
6. Sodium hydroxide (NaOH) (FUJIFILM Wako Pure Chemical Corporation, catalog number: 191-18875)
7. Glycerol (FUJIFILM Wako Pure Chemical Corporation, catalog number: 072-00621)
8. L-Glutamic acid potassium salt (K-Glutamate) (Sigma-Aldrich, catalog number: G1501)
9. Paclitaxel (Taxol) (Sigma-Aldrich, catalog number: T7402)
10. Dimethyl sulfoxide (DMSO) (Nacarai Tesque, catalog number: 09659-14)
11. AFDye 647 NHS (Vector Laboratories, catalog number: FP-1121)
12. Biotin-PEG2-NHS (Tokyo Chemical Industry Co., Ltd., catalog number: B6097)
13. Guanosine 5’-triphosphate (GTP) (Nacarai Tesque, catalog number: 17450-61)
14. PLL-PEG biotin [SuSoS AG, product name: PLL(20)-g[3.5]- PEG(2)/PEG(3.4)- biotin(50%)]
15. Streptavidin (FUJIFILM Wako Pure Chemical Corporation, catalog number: 194-17863)
16. Potassium acetate (KCH_3_COO) (FUJIFILM Wako Pure Chemical Corporation, catalog number: 160-03175)
17. Pluronic F-127 (Sigma-Aldrich, catalog number: P2443)
18. Magnesium Acetate [Mg(CH_3_COO)_2_] (FUJIFILM Wako Pure Chemical Corporation, catalog number: 135-10011)
19. κ-Casein (Sigma-Aldrich, catalog number: C0406)
20. Bovine serum albumin (BSA) (Nacarai Tesque, catalog number: 01863-06)
21. Biotin-BSA (Thermo Fisher Scientific, catalog number: 29130)
22. Adenosine 5’-triphosphate (ATP) (Nacarai Tesque, catalog number: 01072-82)
23. Protocatechuic acid (PCA) (Nacarai Tesque, catalog number: 08521-24)
24. Protocatechuate-3,4-dioxygenase (PCD) (TOYOBO, catalog number: PCO-302)
25. Trolox (FUJIFILM Wako Pure Chemical Corporation, catalog number: 202-17891)
26. Methanol (FUJIFILM Wako Pure Chemical Corporation, catalog number: 134-01833)
27. Ethanol (FUJIFILM Wako Pure Chemical Corporation, catalog number: 057-00541)
28. Hydrogen chloride (HCl) (FUJIFILM Wako Pure Chemical Corporation, catalog number: 087-01076)
29. Sucrose (Nacarai Tesque, catalog number: 30403-55)
30. Vaseline (Nacarai Tesque, catalog number: 36202-05)
31. Lanolin (FUJIFILM Wako Pure Chemical Corporation, catalog number: 128-00115)
32. Paraffin (Nacarai Tesque, catalog number: 26023-65)

**Solutions**

1. BRB80 (see Recipes)
2. High-pH cushion (see Recipes)
3. Labeling buffer (see Recipes)
4. Quench buffer (see Recipes)
5. Low-pH cushion (see Recipes)
6. 1 mM Taxol (see Recipes)
7. Sucrose cushion (see Recipes)
8. Assay buffer (see Recipes)
9. 100 mM Trolox (see Recipes)
10. VALAP (see Recipes)

**Recipes**

1. **BRB80**
  80 mM PIPES (adjust the pH to 6.8 with KOH)
  1 mM MgCl_2_
  1 mM EGTA
2. **High-pH cushion**
  0.1 M HEPES (adjust the pH to 8.6 with NaOH)
  1 mM MgCl_2_
  1 mM EGTA
  60% glycerol
3. **Labeling buffer**
  0.1 M HEPES (adjust the pH to 8.6 with NaOH)
  1 mM MgCl_2_
  1 mM EGTA
  40% glycerol
4. **Quench buffer**
  2× BRB80
  100 mM K-Glutamate (adjust the pH to 7.0 with KOH)
  40% glycerol
5. **Low-pH cushion**
  1× BRB80
  60% glycerol
6. **1 mM Taxol**
  Dissolve Taxol in DMSO.
7. **Sucrose cushion**
  Dissolve 3 g of sucrose in BRB80 with 20 μM Taxol and make up the volume to 10 mL. Rotate the solution at 35 °C overnight.
8. **Assay buffer**
  180 μL of 0.5 M HEPES (adjust the pH to 7.4 with KOH)
  200 μL of 50% glycerol
  50 μL of 1 M KCH_3_COO
  50 μL of 10% Pluronic F-127
  20 μL of 100 mM Mg(CH_3_COO)_2_
  20 μL of 50 mM EGTA
  20 µL of 10 mg/mL κ-Casein
  20 μL of 50 mg/mL BSA
  20 μL of 5 mg/mL biotin-BSA
  1 μL of 1 mM Taxol
9. **100 mM Trolox**
  Mix 430 μL of methanol and 345 μL of 1 M NaOH. Dissolve 100 mg of Trolox in the solution and make up the volume to 4 mL with ddH_2_O. Rotate the solution at 25 °C overnight. Pass the solution through a 0.22 μm filter.
10. **VALAP**
  50 g of Vaseline
  50 g of lanolin
  50 g of paraffin

**Laboratory supplies**

1. Pipette tips
  2–20 μL [Thermo Scientific (QSP), catalog number: TLR102RL-Q]
  10–100 μL [Thermo Scientific (QSP), catalog number: 110RL-NEW]
  100–1,000 μL [Thermo Scientific (QSP), catalog number: T112XLRL-Q]
2. 1.5 mL microtubes (WATSON, catalog number: 131-415C)
3. Thick wall polycarbonate tubes
  0.2 mL (Beckman Coulter, catalog number: 343775)
  1 mL (Beckman Coulter, catalog number: 343778)
  3.5 mL (Beckman Coulter, catalog number: 349622)
4. 0.22 μm filter (MERCK, catalog number: UFC30GV00)
5. Coverslips (THORLABS, catalog number: CG15CH)
6. Microscope slides (MATSUNAMI, catalog number: S1225)
7. Double-sided tape (NICHIBAN, catalog number: NW-25)

## Equipment

1. Micropipette
  0.5–10 μL (Eppendorf, catalog number: 4924000029)
  2–20 μL (Eppendorf, catalog number: 4924000037)
  20–200 μL (Eppendorf, catalog number: 4924000061)
  100–1,000 μL (Eppendorf, catalog number: 4924000088)
2. Ultracentrifuge (Beckman Coulter, model: Optima TLX Ultracentrifuge)
3. Centrifuge rotor (Beckman Coulter, model: TLA-100, TLA120.2, TLA100.3)
4. Water bath (TAITEC, model: CTU-mini, catalog number: 0063288-000)
5. Water bath with sonicator (AS ONE, model: ASU-M)
6. TIRF setup
  Fluorescence microscope (Nikon Instruments, model: ECLIPSE Ti2-E)
  Objective lens (Nikon Instruments, model: CFI Apochromat TIRF 100XC Oil)
  Glass heater (TOKAI HIT, model: TPiD-SQH26-LH)
  Camera (Oxford Instruments, model: Andor iXion life 897)
  Laser (Nikon Instruments, model: Ti2-LAPP illumination system)
  System software (Nikon Instruments, model: NIS-Elements AR software version 5.2)

## Software and datasets

1. ImageJ Fiji [14]

## Procedure

- **Labeling tubulin with biotin or fluorescent dye**
  Prepare biotin-labeled tubulin and fluorescently labeled tubulin to stabilize microtubules on a streptavidin-coated glass surface and visualize them [15].
  1. Make 7 mL of 3 mg/mL tubulin solution by diluting the 7–10 mg/mL purified tubulin solution [13] with BRB80 on ice.
  2. Add 35 μL of 1 M MgCl_2_, 100 μL of 100 mM GTP, and 3.5 mL of glycerol to the solution and gently mix. Incubate the solution in a water bath at 37 °C for 60 min.
  3. Layer 1.75 mL of the solution on 1.4 mL of high-pH cushion in six 3.5 mL thick-wall polycarbonate tubes. Centrifuge at 80,000× *g* for 35 min at 35 °C in the TLA100.3 rotor. High-pH cushion retains non-polymerized tubulin in the supernatant and raises the buffer pH sufficiently for the labeling reaction.
  4. Aspirate the supernatant slowly and wash the interface of the cushion with 1 mL of 37 °C warm labeling buffer twice. Remove the cushion and thoroughly resuspend the pellet in the first tube with 500 μL of 37 °C warm labeling buffer, then sequentially transfer and resuspend in the next tubes up to the sixth tube to combine all pellets into 500 μL of solution.
  5. Add 1 mg of AFDye 647 NHS or Biotin-PEG2-NHS dissolved in DMSO to the solution and vortex it quickly. Incubate the solution in a water bath at 37 °C for 10 min and lightly vortex every 2 min. Add 530 μL of warm (37 °C) quench buffer to the solution and lightly vortex to stop the labeling reaction. Incubate the solution in a water bath at 37 °C for 5 min.
  6. Layer 1 mL of the solution on 1.5 mL of low-pH cushion in a 3.5 mL thick-wall polycarbonate tube. Centrifuge at 80,000× *g* for 20 min at 35 °C in the TLA100.3 rotor. Low-pH cushion retains the free dye in the supernatant.
  7. Aspirate the supernatant slowly and wash the interface of the cushion with warm (37 °C) BRB80 twice. Remove the cushion and thoroughly resuspend the pellet with 500 μL of cold (4 °C) BRB80 on ice.
  8. Transfer the solution into a 1 mL thick-wall polycarbonate tube and centrifuge at 80,000× *g* for 10 min at 4 °C in the TLA120.2 rotor. In this procedure, non-depolymerized tubulin resulting from the labeling process is precipitated and removed.
  9. Take out the supernatant and add 130 μL of 5× BRB80, 2 μL of 1 M MgCl_2_, and 5 μL of 100 mM GTP on ice.
  10. Add 330 μL of glycerol to the solution and gently mix. Incubate the solution in a water bath at 37 °C for 30 min.
  11. Layer 1 mL of the solution on 1.5 mL of low-pH cushion in a 3.5 mL thick-wall polycarbonate tube. Centrifuge at 80,000× *g* for 35 min at 35 °C in the TLA100.3 rotor. Low-pH cushion retains the non-polymerized tubulin in the supernatant.
  12. Aspirate the supernatant slowly and wash the interface of the cushion with warm (37 °C) BRB80 twice. Remove the cushion and wash the interface of the pellet with warm (37 °C) BRB80 twice. Resuspend the pellet with 200 μL of cold (4 °C) BRB80 and incubate on ice for 20 min.
  13. Centrifuge at 80,000× *g* for 10 min at 4 °C in the TLA100 rotor. Finally, non-depolymerized tubulin is precipitated and removed.
  14. Measure the concentration of labeled tubulins using SDS-PAGE. As a control, use unlabeled tubulins with a known concentration.
  15. Aliquot the supernatant in 3 μL portions, freeze in liquid nitrogen, and store at -80 °C.
- **Microtubule polymerization**
  1. To make 25 μL of elongation mix:
    20 μL of unlabeled tubulin (3 mg/mL)
    2 μL of biotin-labeled tubulin (3 mg/mL)
    2 μL of fluorescently labeled tubulin (3 mg/mL)
    1 μL of 100 mM GTP
    The concentrations of biotin-labeled and fluorescently labeled tubulin should be approximately 1/10 to 1/40 of the unlabeled tubulin. In practice, their concentrations should be minimized as much as possible, as excessive labeling can hinder motor movement. It is sufficient for the overall microtubule fluorescence on the glass to be just barely detectable under the microscope.
  2. Incubate the mix in a water bath at 37 °C for more than 20 min. Microtubules that are approximately 10–20 μm in length are polymerized after 20 min of incubation. Incubating for around 45 min results in microtubules approximately 50 μm long. However, microtubules longer than 50 μm are generally unnecessary, as they often extend beyond the field of view in TIRF microscopy.
  3. Add 25 μL of 40 μM Taxol to the mix and incubate at 37 °C for more than 15 min.
  4. Layer the mix on 150 μL of sucrose cushion in a 0.2 mL thick-wall polycarbonate tube. Centrifuge at 50,000× *g* for 10 min at 35 °C in the TLA-100 rotor. Sucrose cushion retains the non-polymerized tubulin in the supernatant.
  5. Aspirate the supernatant slowly and wash the interface of the cushion with warm (37 °C) BRB80 twice. Remove the cushion and thoroughly resuspend the pellet with 50 μL of BRB80 with 20 μM Taxol.
  6. Store the microtubules at room temperature and protect from light.
- **TIRF single-molecule assay**
  1. Wash glass chamber.
    - Acid wash the coverslips with HCl for 24 h and rinse with ddH_2_O.
    - Fill the container with ddH_2_O and sonicate in a water bath at 42 kHz three times for 30 min each.
    - Fill the container with 50%, 70%, and 95% ethanol sequentially, and sonicate in a water bath at 42 kHz for 30 min for each concentration.
    - Fill the container with 95% ethanol and store at room temperature.
  2. Make a glass chamber.
    - Burn both sides of the coverslip with a gas burner to volatilize the ethanol (Figure 1A).
    - Attach the coverslip to a glass slide using two pieces of double-sided tape at the top and bottom, creating open slits on both sides (Figure 1B). The channel width is approximately 3 mm. If the observation time in one channel is short enough to prevent evaporation in the second channel (approximately 1–3 min), two channels can be prepared on a single coverslip to quickly examine two conditions (Figure 1C).
      **Figure 1.**
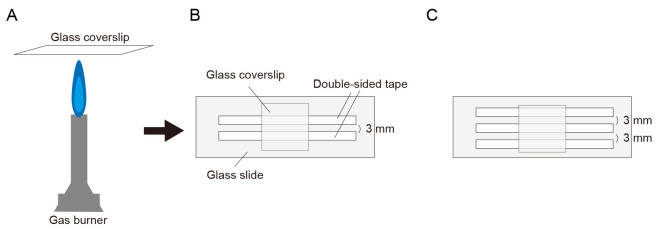
Illustration of glass slide chamber. (A) Burning both sides of a coverslip with a gas burner. (B) Making glass slide chamber using double-sided tape. (C) Preparing two channels for examining and comparing two conditions.
    - Flow in 10 μL of 0.5 mg/mL PLL-PEG-biotin into the chamber and let it sit for 5 min. PLL-PEG coats the glass surface and prevents the non-specific binding of proteins.
    - Flow in 10 μL of 0.5 mg/mL streptavidin into the chamber and let it sit for 2 min.
    - Wash out the unbound streptavidin with 2 × 20 μL of BRB80.
    - Flow in 10 μL of labeled microtubules at a 1/50 dilution (in BRB80 with 20 μM Taxol) into the chamber and let it sit for more than 5 min.
    - Wash out the unbound microtubules with 2 × 20 μL of assay buffer.
    - To make 50 μL of observation mix:
      29 μL of assay buffer
      16 μL of ddH_2_O
      1 μL of 10 pM to 10 nM purified kinesin (depending on the type of kinesin)
      1 μL of 100 mM ATP
      1 μL of 100 mM PCA
      1 μL of 2.5 mM PCD
      1 μL of 100 mM Trolox
      PCA, PCD, and Trolox serve as an oxygen scavenging system.
    - Flow in 10 μL of the observation mix into the chamber.
  3. Observe the single-molecule motility of motor proteins under the TIRF microscope at room temperature (24 ± 1 °C). In this protocol, 15 mW 488 nm, 561 nm, and 647 nm excitation lasers are used. Videos 1–3 are examples of TIRF single-molecule assays of KIF1A/UNC-104 (kinesin-3). Video 1 shows the motility of artificially dimerized truncated KIF1A using the leucine zipper domain [KIF1A(1-393)LZ]. The red fluorescent protein mScarlet I was attached to its C-terminal. In the first frame, an image of microtubules was taken using 640 nm excitation (laser strength: 9%). In the remaining frames, images of KIF1A(1-393)LZ were taken using 561 nm excitation (laser strength: 5%) at 10 frames per second (fps) and saved in the nd2 file format. In Video 1, images from two color channels were merged. Videos 2 and 3 show the motility of truncated UNC-104 [UNC-104(1-653)] and its mutant [UNC-104(1-653)(E412K)], respectively. The green fluorescent protein sfGFP was attached to their C-terminals. In the first frame, an image of microtubules was taken using 640 nm excitation (laser strength: 9%). In the remaining frames, images of UNC-104 were taken using 488 nm excitation (laser strength: 5%) at 10 fps and saved in the nd2 file format. In Videos 2 and 3, images from two color channels were merged.
    **Video 1.**
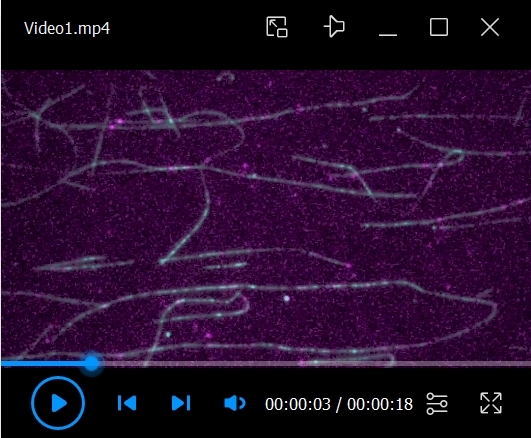
Example of total internal reflection fluorescence (TIRF) single-molecule assay of KIF1A(1-393)LZ labeled by mScarlet I. Video is played at 5× speed. Scale bar shows 10 μm.
    **Video 2.**
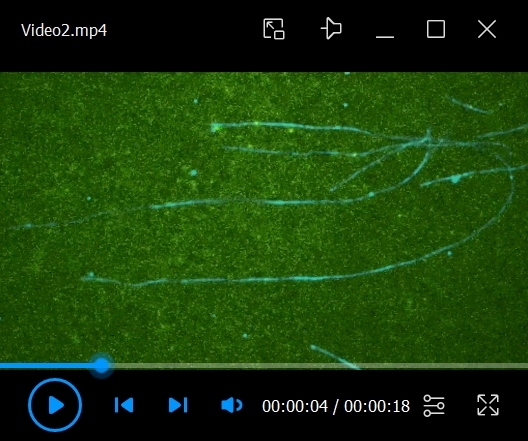
Example of total internal reflection fluorescence (TIRF) single-molecule assay of UNC-104(1-653) labeled by sfGFP. Video is played at 5× speed. Scale bar shows 10 μm.
    **Video 3.**
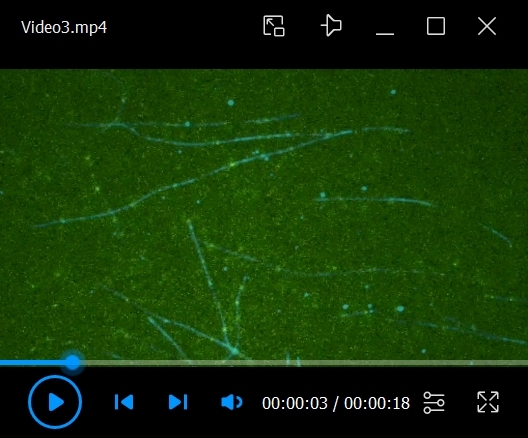
Example of total internal reflection fluorescence (TIRF) single-molecule assay of UNC-104(1-653)(E412K) labeled by sfGFP. Video is played at 5× speed. Scale bar shows 10 μm.

## Data analysis

1. Figure 2 illustrates the principle of a TIRF single-molecule assay. Fluorescent microtubules are fixed onto a PLL-PEG biotin and streptavidin-coated coverslip. An aliquot of kinesins labeled with fluorescent proteins is introduced into the chamber. The movements of the fluorescently labeled kinesins are observed as they move continuously in a unidirectional manner.
  **Figure 2.**
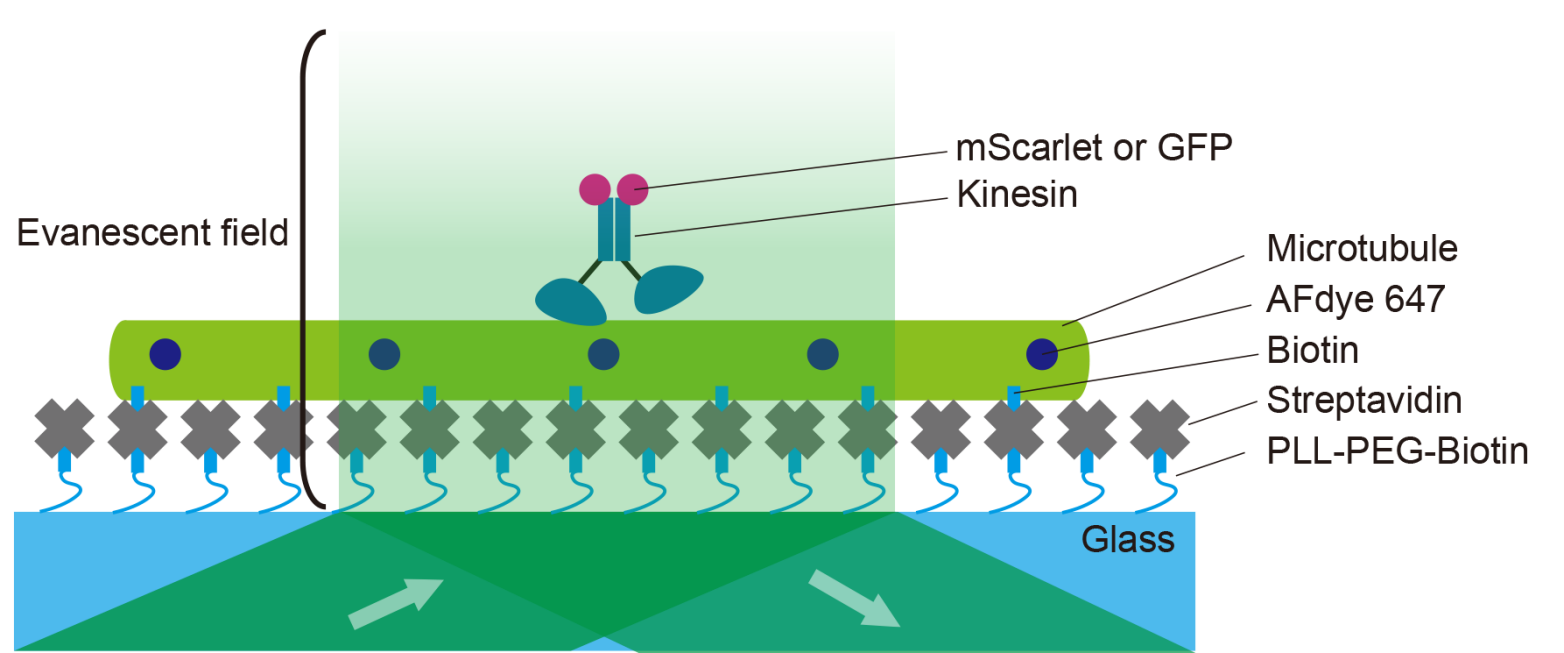
Illustration of a single-molecule assay. The coverslip is coated with PLL-PEG-biotin followed by streptavidin. Microtubules are then flowed into the chamber and fixed onto the coverslip. Fluorescently labeled kinesins are introduced into the chamber. Kinesin motility is observed under a total internal reflection fluorescence (TIRF) microscope.
2. ImageJ Fiji was used to create kymographs for the analysis of kinesin. Import the nd2 file into ImageJ Fiji. Use the *Segmented Line* tool to draw a line along the microtubule in the 640 nm channel. Create a kymograph using the *KymographBuilder* plugin. Figure 3 illustrates the principle of kymograph analysis. Figure 3A shows a series of images taken at different time points in Video 1, with kinesins moving from left to right as time progresses. Similarly, the kymograph is a two-dimensional representation, where the x-axis represents time, and the y-axis represents the spatial position along the selected microtubule (Figure 3B). In the kymograph, moving objects appear as diagonal lines (Figure 3B).
  **Figure 3.**
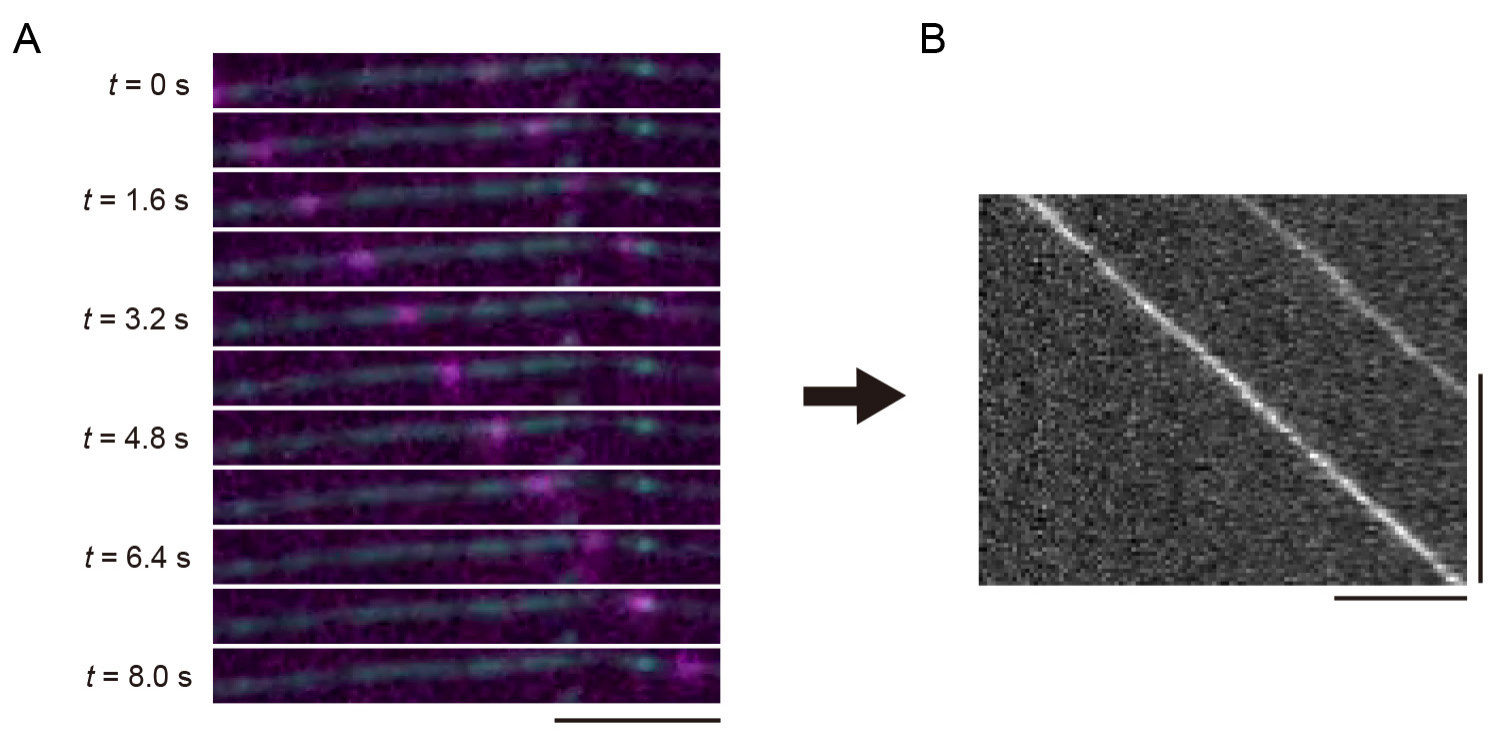
Unidirectional movements of kinesins and its kymograph. (A) Sequential frames showing representative single-kinesin movements from Video 1. A scale bar shows 5 μm. (B) The kymograph of (A). Horizontal and vertical bars show 5 μm and 5 s, respectively.
3. We demonstrate the analysis of the TIRF single-molecule assay of UNC-104. UNC-104 is a kinesin-3 family protein that transports synaptic vesicle precursors in *C. elegans* axons. UNC-104(1-653), a cargo-binding domain deletion mutant, is strongly autoinhibited and rarely bound to microtubules (Video 2). In contrast, UNC-104(1-653)(E412K), an autoinhibition-disrupted mutant, is frequently bound to microtubules (Video 3). To quantify its extent of activation, we analyzed velocity, run length, and landing rate from their kymographs (Figure 4A). The slope of the diagonal lines indicates the velocity of the movement: steeper slopes represent faster movement, while shallower slopes indicate slower movement. The distance along the x-axis between the attachment and detachment points of motors on the microtubule represents the run length. The landing rate is calculated by dividing the number of motor attachments on the microtubule by the observation time and length of the microtubule. The run length and landing rate of UNC-104(1-653)(E412K) significantly increased compared to UNC-104(1-653), but their velocities remained the same [4] (Figure 4B–D).
  **Figure 4.**
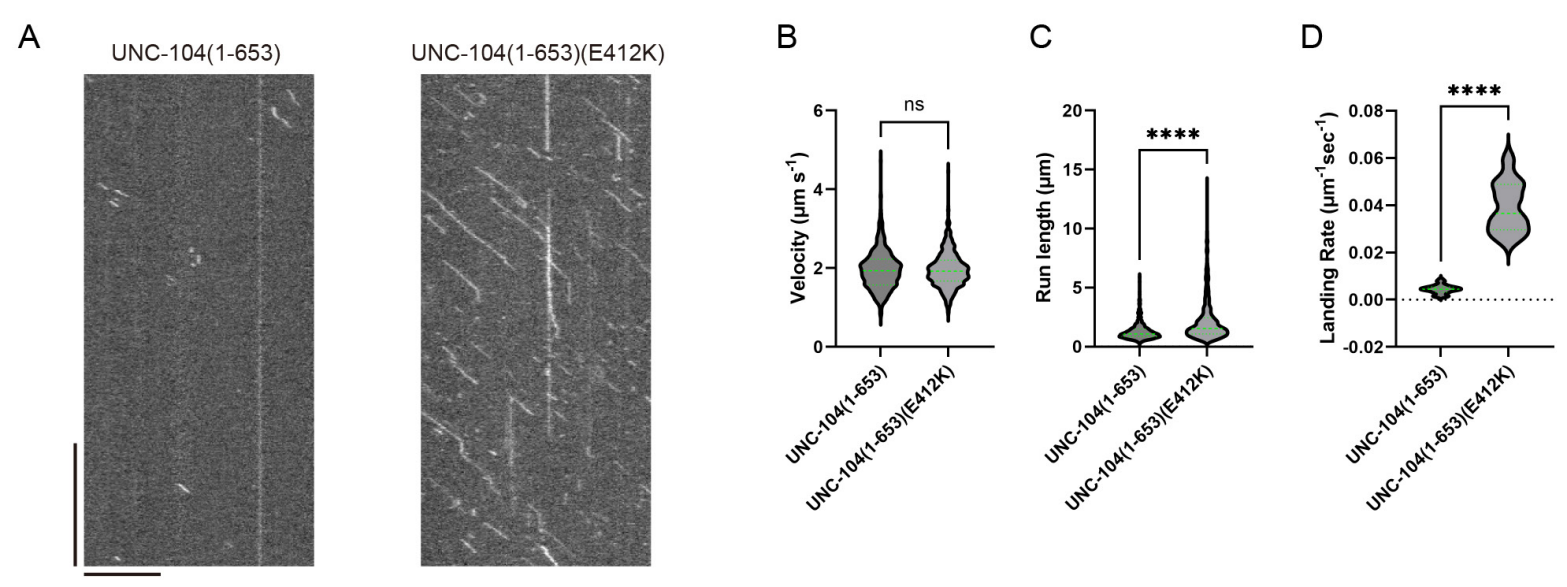
Representative results. (A) Representative kymographs of UNC-104(1-653) and UNC-104(1-653)(E412K) from Video 2 and Video 3, respectively. Horizontal and vertical bars show 10 μm and 10 s, respectively. (B) The velocities of UNC-104(1-653) and UNC-104(1-653)(E412K) are plotted as violin graphs. Green bars represent mean ± SD. Student’s t-test, ns, p = 0.7 and statistically not significant. (C) The run lengths of UNC-104(1-653) and UNC-104(1-653)(E412K) are plotted as violin graphs. Green bars represent the median value and interquartile range. Mann-Whitney U test. ****, p < 0.0001. (D) The landing rates of UNC-104(1-653) and UNC-104(1-653)(E412K) are plotted as violin graphs. Green bars represent the median value. Mann-Whitney U test. ****, p < 0.0001. The values in (B)–(D) are identical to those reported in Kita et al. [4]; see the reference for further details.

*Notes:*

- *Polymerized microtubules can be stored at room temperature for 2–3 weeks. Microtubules are depolymerized at low temperatures and should not be refrigerated.*
- *Kinesin proteins must be stored at -80 °C.*
- *Single-molecule assays for kinesins are typically performed at room temperature (24 ± 1 °C). The chamber temperature can be regulated using a glass heater. The temperature dependency varies depending on the type of motor [16].*

## Validation of protocol

This protocol or parts of it has been used and validated in the following research article(s):

- Kita et al. [4]. Comparative analysis of two Caenorhabditis elegans kinesins KLP-6 and UNC-104 reveals a common and distinct activation mechanism in kinesin-3 eLife (Figure 1D, Figure 2, Figure 3C–E, Figure 4D–F, Figure 5D–G, and Figure 7D–G).]

## General notes and troubleshooting

**General notes**

This protocol can be applied to analyze other kinesin superfamily proteins and dynein motor proteins from different species. Some motor proteins may be very slow. In that case, one needs to adjust the frame rate. 100 ms/frame for 1 min is the starting point. We sometimes observe very slow motors at 2 s/frame for 10 min. To prevent evaporation in the channels, it is recommended to seal the channels with VALAP during observation.

**Troubleshooting**

Problem 1: Microtubules are short.

Possible causes: Excessive pipetting and/or insufficient incubation time.

Solutions: Reduce pipetting or use gentle tapping instead. Extend the incubation time at 37 °C to 45 min.

Problem 2: Microtubules are not stabilized on the glass and/or are not visualized with fluorescence.

Possible causes: Insufficient biotin-labeled tubulin and/or fluorescently labeled tubulin.

Solutions: Increase the concentration of biotin-labeled and/or fluorescently labeled tubulin during polymerization. However, note that excessive labeling can impede kinesin movement. It is sufficient for the overall microtubule fluorescence on the glass to be just barely detectable under the microscope.

Problem 3: Kinesins move very slowly or do not move.

Possible causes: Excessive labeling of microtubules.

Solutions: Reduce the concentration of biotin-labeled and/or fluorescently labeled tubulin during polymerization. KIF1A(1-393)LZ can serve as a reliable positive control to verify the conditions. In our setup, it moves at approximately 1.5 μm/s along microtubules.
